# Vitamin B12 Deficiency among Metformin Treated Type 2 Diabetic Mellitus Patients Visiting the Department of Medicine of a Tertiary Care Centre

**DOI:** 10.31729/jnma.8340

**Published:** 2023-11-30

**Authors:** Anil Yadav, Sabita Jyoti, Ram Kumar Mehta, Surya Bahadur Parajuli

**Affiliations:** 1 Department of Medicine, Birat Medical College Teaching Hospital, Biratnagar, Morang, Nepal; 2 Department of Community Medicine, Nepalgunj Medical College, Nepalgunj, Banke; 3 Department of Community Medicine, Birat Medical College Teaching Hospital, Biratnagar, Morang, Nepal

**Keywords:** *diabetic neuropathy*, *metformin*, *type 2 diabetes*, *vitamin B12*

## Abstract

**Introduction:**

About 424.9 million people worldwide are affected by Diabetes mellitus. Prevalence among people 20-79 years old in Nepal was 4% in 2017. It is associated with microvascular and macrovascular complications such as peripheral neuropathy leading to risk of foot ulcers and amputation, and impaired sensation in their feet. The study aimed to find the prevalence of vitamin B12 deficiency among metformin-treated type 2 diabetic patients visiting the Department of Medicine of a tertiary care centre.

**Methods:**

A descriptive cross-sectional study was conducted in a tertiary care centre between 24 May 2021 to 24 May 2022 after obtaining ethical approval from the Institutional Review Committee. Patients who visited the Department of Medicine and gave informed consent were included in the study. Patients with underlying comorbidities were excluded from the study. A convenience sampling method was used. The point estimate was calculated at a 95% Confidence Interval.

**Results:**

Among 330 patients, vitamin B12 deficiency was seen in 33 (10%) (6.76-13.24, 95% Confidence Interval). Among them, 27 (81.82%) were male and 6 (18.18%) were female.

**Conclusions:**

The prevalence of vitamin B12 deficiency was found to be higher than other studies done in similar settings.

## INTRODUCTION

Diabetes mellitus (DM) is a metabolic disorder affecting about 424.9 million people worldwide with the majority of patients about 90% having type 2 Diabetes.^1^ International Diabetes Federation (IDF) reported the prevalence of DM among people 20-79 years 4% in Nepal in 2017 and expected to rise by 6.1% by 2045.^2^

Metformin is the most prescribed anti-diabetic drug for Type 2 Diabetes mellitus and is considered a cornerstone in the treatment of Type 2 DM.^3^ It is effective and safe, inexpensive but one of the side effects of metformin is to reduce vitamin B12 status. Vitamin B12 deficiency is underdiagnosed and undertreated.^4,5^ Severe deficiency (pernicious anaemia) can result in macrocytic anaemia, peripheral neuropathy, and mental psychiatric changes while a decrease in level starts as early as the fourth month.^6,7^

The study aimed to find the prevalence of vitamin B12 deficiency among metformin-treated type 2 DM patients visiting the Department of Medicine of a tertiary care centre.

## METHODS

A descriptive cross-sectional study was conducted at the Department of Medicine of Birat Medical College Teaching Hospital, Biratnagar, Morang, Nepal from 24 May 2021 to 24 May 2022. Ethical approval was taken from the Institutional Review Committee of the same institute (Reference number: IRC-PA-131/2077-78). Patients who visited the Department of Medicine and gave informed consent were included in the study. Patients with underlying comorbidities (tuberculosis, type 1 DM) were excluded from the study. A convenience sampling method was used. The sample size of the study was calculated using the formula:


$$\begin{array}{ll} \text{n} & ={\text{Z}}^{2}\times \frac{\text{p}\times \text{q}}{{\text{e}}^{\text{2}}}\\ & =1.96^{2}\times \frac{0.50\times 0.50}{0.06^{2}}\\ & =267 \end{array}$$

Where,

n = minimum required sample size
Z = 1.96 at 95% of Confidence Interval (CI)
p = prevalence taken as 50% for maximum sample size calculation
q = 1-p
e = margin of error, 6%

The calculated minimum required sample size was 267. However, 330 patients were included in this study.

After obtaining approval and permission from the Department and written informed consent patients and family members. Several parameters such as vitamin B12 levels and severity of peripheral neuropathy were assessed by using a predesigned proforma. Also, the duration of diabetes, duration of metformin usage, dietary history, and HbA1c levels were assessed.

Data were entered using Microsoft Excel 2010 and analysis was performed using IBM SPSS Statistics version 21.0. The point estimate was calculated at a 95% CI.

## RESULTS

Among 330 patients with type 2 DM on metformin, vitamin B12 deficiency was seen in 33 (10%) (6.76-13.24, 95% CI). Among them, 27 (81.82%) were male and 6 (18.18%) were female (Table 1).

**Table 1:** Socio-demographic distribution of patients with vitamin B12 deficiency (n= 33).

Variables	n (%)
**Gender**	
Male	27 (81.82)
Female	6 (18.18)
**Age (years)**	
<60	27 (81.82)
≥60	6 (18.18)
**Residency**	
Urban	30 (90.91)
Rural	3 (9.09)

A total of 16 (48.48%)had optimal weight and 17(51.52%) were overweight (Table 2).

**Table 2:** Dietary habits among patients with vitamin B12 deficiency (n = 33).

Variables	n (%)
**Smoking**	
Current smoker	5 (15.15)
Past smoker	18 (54.55)
Non-smoker	10 (30.30)
**Dietary habit**	
Vegetarian	3 (9.09)
Non-vegetarian	30 (90.91)
**Alcohol**	
Consumer	1 (3.03)
Non-consumer	24 (72.73)
Quitted	8 (24.24)

## DISCUSSION

The prevalence of vitamin B12 deficiency was seen among 33 (10%). This prevalence is higher compared to National data from the International Dairy Federation in Nepal which showed 4%.^2^ The finding from this study has shown lower vitamin B12 levels among 20.6% of patients, and an increased risk of borderline or frank B12 deficiency with metformin use which is similar to the previous study in 27 centers in the USA.^8^ These findings also revealed long-term use of metformin in the Diabetes Prevention Program Outcomes Study was associated with biochemical B12 deficiency and anaemia.^8^ In the Nhanes Survey 1999 to 2006, it was seen that 5.8% of diabetic patients had definite Vitamin B12 deficiency and 16.2% of diabetics on metformin had borderline Vitamin B12 deficiency on metformin therapy which is similar to the previous study.^9^

Low Hb levels with higher Mean corpuscular volume values were observed in metformin-treated patients having low Vitamin B12 levels. This finding is consistent with our existing knowledge of Vitamin B12 deficiency being an important cause of macrocytic anaemia.^10^ Our finding shows vitamin B12 deficiency to be more in males than in females. In a cross-sectional study conducted among the healthy Israeli population men were also susceptible to vitamin B12 deficiency. This can be explained by neither diet habits nor estrogen effects. Genetic variations are therefore hypothesized to play a role.^11^

The triglycerides were raised in 87.2% of patients and a similar study reports vitamin B12 was high and independently associated with triglycerides in Indian and European type 2 diabetic patients on metformin which is similar to our study.^12^ Vitamin B12 deficiency was seen in 5.4% of patients having abnormal blood pressure. A similar study reported that in essential hypertension, there is a continuous negative correlation of serum B12 on the contrary.^13^

## CONCLUSIONS

The prevalence of vitamin B12 deficiency was found to be higher than other studies done in similar settings.
